# Homozygosity in any HLA locus is a risk factor for specific antibody production: the taboo concept 2.0

**DOI:** 10.3389/fimmu.2024.1384823

**Published:** 2024-05-22

**Authors:** Henry Loeffler-Wirth, Claudia Lehmann, Nils Lachmann, Ilias Doxiadis

**Affiliations:** ^1^ Interdisciplinary Centre for Bioinformatics (IZBI), Leipzig University, Leipzig, Germany; ^2^ Laboratory for Transplantation Immunology, University Hospital Leipzig, Leipzig, Germany; ^3^ Institute for Transfusion Medicine, H & I Laboratory, Charité – Universitätsmedizin Berlin, Corporate Member of Freie Universität Berlin and Humboldt Universitätzu Berlin, Berlin, Germany

**Keywords:** homozygosity, organ transplantation, HLA specific antibodies, risk stratification, high resolution typing, entropy

## Abstract

**Objective:**

In a cooperative study of the University Hospital Leipzig, University of Leipzig, and the Charité Berlin on kidney transplant patients, we analysed the occurrence of HLA-specific antibodies with respect to the HLA setup of the patients. We aimed at the definition of specific HLA antigens towards which the patients produced these antibodies.

**Methods:**

Patients were typed for the relevant HLA determinants using mainly the next-generation technology. Antibody screening was performed by the state-of-the-art multiplex-based technology using microspheres coupled with the respective HLA alleles of HLA class I and II determinants.

**Results:**

Patients homozygous for HLA-A*02, HLA-A*03, HLA-A*24, HLA-B*07, HLA-B*18, HLA-B*35, HLA-B*44, HLA-C*03, HLA-C*04, and HLA-C*07 in the class I group and HLA-DRB1*01, HLA-DRB1*03, HLA-DRB1*07, HLA-DRB1*15, HLA-DQA1*01, HLA-DQA1*05, HLA-DQB1*02, HLA-DQB1*03(7), HLA-DQB1*06, HLA-DPA1*01, and HLA-DPB1*04 in the class II group were found to have a significant higher antibody production compared to the heterozygous ones. In general, all HLA determinants are affected. Remarkably, HLA-A*24 homozygous patients can produce antibodies towards all HLA-A determinants, while HLA-B*18 homozygous ones make antibodies towards all HLA-B and selected HLA-A and C antigens, and are associated with an elevation of HLA-DRB1, parts of DQB1 and DPB1 alleles. Homozygosity for the HLA class II HLA-DRB1*01, and HLA-DRB1*15 seems to increase the risk for antibody responses against most of the HLA class I antigens (HLA-A, HLA-B, and HLA-C) in contrast to HLA-DQB1*03(7) where a lower risk towards few HLA-A and HLA-B alleles is found. The widely observed differential antibody response is therefore to be accounted to the patient’s HLA type.

**Conclusion:**

Homozygous patients are at risk of producing HLA-specific antibodies hampering the outcome of transplantation. Including this information on the allocation procedure might reduce antibody-mediated immune reactivity and prevent graft loss in a patient at risk, increasing the life span of the transplanted organ.

## 1 Introduction

The molecules of the human leucocyte antigen system (HLA) play a pivotal role in immune recognition, and response. Their role in pregnancy, transfusion, and transplantation has been readily described (1). HLA molecules are receptor molecules for peptides presented to immune cells. Finally, they are the targets of immune response upon solid organ and stem cell transplantation. It is generally accepted that the formation of antibodies towards an allograft’s HLA leads to severe consequences for the graft and the patient (2). The number of individual alleles grows exponentially, reaching >38,000 to date (3). Methodologically, molecular typing by next-generation sequencing technology (NGS) and the bead-bound HLA molecules for antibody screening replaced earlier outdated techniques (4–7).

Homozygosity can, on the one side, be considered as injurious in case of recessive genes, leading to diseases such as cystic fibrosis, endocrinological disorders, sickle cell anaemia, or other harmful mutations leading to incurable situations (8). On the other side, homozygosity can be beneficial, e.g., in rhesus factor compatibility, and in all unmutated genes, homozygosity is deemed positive (8).

It is, however, still controversial whether homozygosity for any of the HLA loci is beneficial for the individual or the population (9–13). This information was retrieved using mostly low-resolution typing, with the exception of Hönger et al., who elaborated on the production of HLA antibodies in pregnancy. While several reports advocating that partner selection and mating are in favour of a still, presumably theoretical heterozygous advantage, field results do not support this hypothesis (14, 15). In viral infections such as HIV or SARS-CoV-2, homozygous patients are at risk compared to heterozygous individuals (16, 17).

A proportion of patients on the national and international transplantation solid organ waitlists are homozygous for the HLA loci HLA-A, HLA-B, and HLA-DRB1, which are deemed transplantation relevant: in the allocation procedure, these patients receive special attention upon the availability of a suitable organ donor (18). Earlier studies showed that these patients accumulate on the waiting list and have a decreased graft survival rate and higher degree of sensitisation, which hampers the opportunity to be offered a suitable re-transplant (19). In general, the special attention to homozygous patients is restricted to mainly fully homozygous patients, as introduced in several organ procurement organisations. As a surrogate to transplantation results, we concentrate on the occurrence of specific antibodies in a subcohort of patients with a homozygosity on a single or multiple loci. Earlier, we reported that specific patient HLA combination led to a poorer graft survival, termed as the taboo concept (20). Here, we propose further development of that concept. The updated concept points to the probability of antibody production for single or multiple loci homozygous patients. In the present observational study, we concentrated on the occurrence of homozygosity of one or more of the eight HLA loci tested, and on their influence on the production of alloantibodies. These loci are HLA-A, HLA-B, HLA-C, HLA-DRB1, HLA-DQA1, HLA-DQB1, HLA-DPA1, and HLA-DPB1. We did not consider the loci HLA-DRB3, HLA-DRB4, and HLA-DRB5 because they are in tight linkage disequilibrium to and expressed in linkage with HLA-DRB1 genes.

## 2 Materials and methods

### 2.1 Think different

The cooperation between the Transplantation Immunology (HLA) laboratories of the University Hospital of Leipzig and the University Hospital of the Charité, Berlin, together with the bioinformatics at the Interdisciplinary Centre for Bioinformatics (IZBI), were termed “think different”. This group developed the “think different” concept: in essence, transplantation relevant data such as HLA typing and screening for HLA antibody results using defined commercial lots are retrieved, controlled, and used for machine learning analyses (21–23). The goal is to predict alloimmunisation towards HLA according to the patient’s immunogenetical background.

### 2.2 Sample acquisition

HLA typing and antibody data were retrospectively collected from patients in need of solid organ or haematopoietic stem cell transplant and, additionally, typing data from potential donors. In total, 65,169 individuals are contained in our data set.

High-resolution immunogenetic molecular typing for HLA-A, HLA-B, HLA-C, HLA-DRB1, HLA-DRB3/4/5, HLA-DQA1, HLA-DQB1, HLA-DPA1, and HLA-DPB1 was performed by next-generation typing (NGS): DNA was isolated from anticoagulated peripheral blood samples according to the manufacturer’s recommendations (QIAamp DNA Blood Mini and EZ1 DNA Blood 350 µl Kit, Qiagen, Hilden, Germany). HLA high-resolution typing was performed using commercial test kits, the Alltype NGS 11-Loci (One Lambda, West Hills, CA, USA), and AlloSeq® Tx 17 (CareDx, Brisbane, CA, USA). The sequencing was performed on a MiSeq or MiniSeq Sequencing device (Illumina, San Diego, CA, USA), strictly following the manufacturer’s recommendations. Both laboratories perform the HLA NGS typing according to standards issued by the European Federation for Immunogenetics (EFI). High-resolution typing is available for 29,581 individuals. For the remaining 35,588 individuals, low-resolution typing was performed by quantitative polymerase chain reaction (qPCR), sequence-specific primers (PCR-SSP), or sequence-specific oligonucleotides (PCR-SSO) using assays from One Lambda (West Hills, CA, USA), or Care DX (CareDx, Brisbane, CA, USA).

HLA antibody data are available for 7,234 patients. The majority of them was listed for kidney transplantation (≈66%), followed by heart (10%), liver (7%), lung (7%), and bone marrow (7%), while the time of blood sampling was split into before (63% of all patients) and after (37%) transplantation/transfusion.

Data on HLA class I (HLA-A, HLA-B, and HLA-C) and HLA class II (HLA-DRB1, HLA-DQA1, HLA-DQB1, HLA-DPA1, and HLA-DPB1) antibodies were generated following a step-by-step diagnostics scheme specified by the European Federation for Immunogenetics (EFI): The involved laboratories are obliged to follow this standard, which defines conditions (e.g., diagnosis and previous diagnostics) under which HLA class I and/or class II antibody abundances are to be measured. In consequence, sample numbers differ between the two classes: class I antibody data are available for 4,515 individuals and class II antibodies for 5,793 individuals (overlap of 3,074 individuals). Antibody identification was performed using Luminex Single Antigen Bead assays (One Lambda, West Hills, CA, USA) LSA1A04 lots 10, 11, 12, and LSA2A01 lots 11, 12, 13 from the time period 2016–2021, respectively. Assays have been performed according to the manufacturer’s instructions.

Sample and feature numbers of antibody and typing data subsets are shown in the schematic of our analysis workflow (**Supplementary Figure 1**). Individual data on antibody abundances and homozygosity are provided as **Supplementary Table 1**.

### 2.3 Homozygosity prevalence

We counted the number of homozygous persons for each locus regardless of the specific homozygous allele. This locus-wise homozygosity information serves as stratification for subsequent antibody specificity evaluation. For more detailed analyses, we also extracted allele-wise homozygosity, regarding only a particular allele. Thereby, we only considered alleles with more than 10 homozygous individuals.

### 2.4 Measures for antibody abundance and specificity

Our statistical analysis workflow for the antibody data involve a series of successive analysis steps as illustrated in **Supplementary Figure 1**. It was built on the foundation of the two complementary statistical measures prevalence and entropy to describe overall abundance of antibodies in a patient’s serum and their specificity, i.e., if there is a broad immunisation as reflected in a uniform distribution of antibody MFI values, or if it shows few although very high MFI peaks in the serum (see example antibody profiles in **Supplementary Figure 1C**).

The first measure is the fraction of antibody beads of a person that exceeds an MFI value of 1,500. This cutoff is recommended by the manufacturer of the Antigen Bead Assays and is commonly considered as threshold of antibody production (24). This measure characterises the range of antibodies produced by each person and locus and is indicated as *%MFI< 1,500* throughout the article and figures.

The second measure is the normalised Shannon entropy (25) and considers the distribution of antibody MFI values in a person: this measure originates in information theory and ranges from 0 for persons with only one abundant antibody to 1 for persons with entirely uniform antibody MFI values (26). For one person, entropy is computed as $H=-\sum_{i}^{N}p_{i}\cdot log_{2}(p_{i})$ , where N is the total number of antibody beads, and p_i_ is the relative MFI of each bead: $p_{i}=MFI_{i}/\sum_{i=1}^{N}MFI_{i}$ . Entropy is finally normalised to allow for comparison between different loci, which differ in N: $H_{norm}=H/log_{2}\left(N\right)$ .

In our workflow, we make use of both measures because both aspects of an individual’s antibody repertoire, abundance, and specificity are crucial in the context of transplantation and risk assessment of homozygosity.

### 2.5 Specificity profiling and quadrant segmentation

For a selected locus, a person’s antibody spectrum can be characterised by the two measures *%MFI<1,500* and normalised entropy *H_norm_.* These values are computed for all individuals in the study and subsequently plotted into a two-dimensional coordinate system using *%MFI<1,500* as x-coordinate and *H_norm_* as y-coordinate (**Supplementary Figure 1D**). The resulting biplot is then segmented into four quadrants by dichotomisation of the *%MFI<1,500* and the *H_norm_* values of all persons, respectively, using the slope-weighted average as threshold (27). The defined thresholds are 49.5% for the former and 0.579 for the latter measure, partitioning the coordinate system in persons with low/high *%MFI< 1,500* along the x-coordinate and in persons with low/high *H_norm_* along the y-coordinate.

The so-determined quadrants are populated by persons with specific characteristics of their antibody profile (see example profiles in **Supplementary Figure 1E**): the bottom left quadrant collects persons with low *%MFI< 1,500* and low *H_norm_* values, representing persons with overall low antibody MFI levels, however, with MFI spikes of few specific bead reactions.

The top left quadrant refers to persons with low *%MFI< 1,500* but high *H_norm_* values. This means that the distribution of antibody MFI values is rather uniform, but on a low level mainly below the MFI threshold of 1,500. In other words, the corresponding persons show low antibody levels against a broad range of HLA alleles.

The top right quadrant is the most interesting in the context of immunisation, as it is populated by persons with both high *%MFI > 1,500* and high *H_norm_* values. They are characterised by uniformly high antibody levels against most HLA alleles in the data.

The bottom right quadrant is empty, as no persons show antibody profiles with a high number of beads exceeding MFI of 1,500 accompanied by low entropy, indicating even higher MFI spikes for few beads. Such characteristic is implausible due to technical limitations such as specificity and saturation effects.

The distribution of all individuals in the biplot across the quadrants is visualised in terms of pie charts and as barplots grouped according to homozygosity of the individuals (**Supplementary Figure 1F**). Individuals in the top right quadrant (High %MFI>1,500 and High entropy) show high antibody levels against a broad range of alleles, thus being at risk for organ rejection. The prevalence of this quadrant is screened in individuals with different homozygous alleles.

## 3 Results

### 3.1 Homozygosity prevalence in the study population

**Figure 1:**
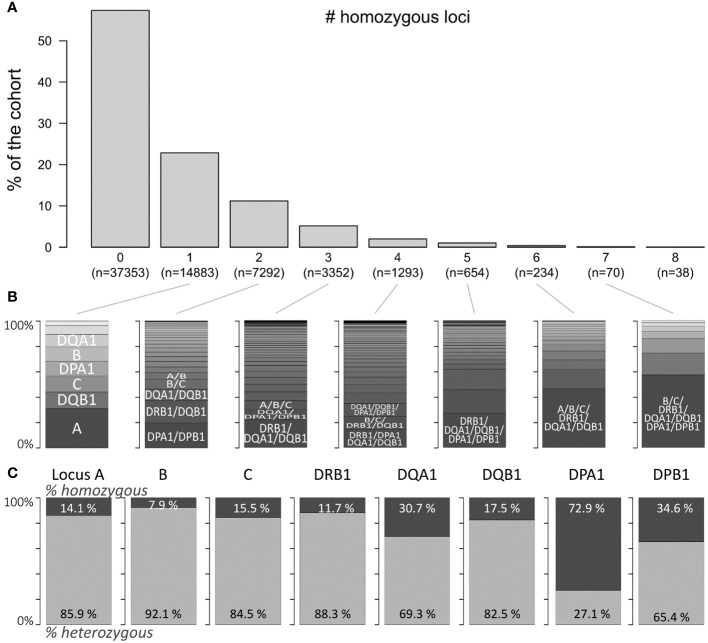
Prevalence of HLA locus homozygosity in the cohort: **(A)** proportion of persons with zero to eight homozygous loci of the eight loci investigated. Corresponding absolute numbers are given below the bars. **(B)** Distributions of homogenous loci combinations in the corresponding subpopulations indicated in **(A)**. **(C)** Proportions of homozygous and heterozygous persons with regard to each locus.

**Figure 1** depicts the fraction of individuals in the study with homozygosity in one or more of the eight HLA loci included in the study (**Figure 1A**; the respective absolute numbers are given below the bars). Majority (≈57%) of the individuals in the study cohort showed no homozygosity at any HLA locus, and proportions of persons with one or more homozygous loci decrease monotonically as expected: the degree of homozygosity per individual differed from one to eight (22.8%–0.06%, respectively). For each degree, we investigated which combinations of homozygous alleles are prevalent in our study cohort (**Figure 1B**). For example, the locus HLA-A was most frequently found homozygous in individuals with a single homozygosity for HLA (≈31%), followed by homozygous DQB1 and C loci (both ≈13%). For a homozygosity degree of 2, joint homozygosity of DPA1 and DPB1 was most frequently found (≈19%); further prevalent combinations were DRB1 and DQB1 (≈18%), and DQA1 and DQB1 (≈9%).

Locus-wise homozygosity ranged between 7.9% for HLA-B and 72.9% for DPA1 (**Figure 1C**). This outcome was expected, since HLA-B is the most polymorphic locus and HLA-DPA1 the least (3). Please note here that these proportions refer to the total number of individuals with typing available for the respective locus, which ranges between more than 60,000 typing results of the HLA-B locus and approximately 10,000 for HLA-DPA1 and HLA-DPB1 loci due to data acquisition guidelines of the participating transplant centres (see **Supplementary Figure 2**).

### 3.2 Class I antibody specificity in homozygous and heterozygous individuals

**Figure 2:**
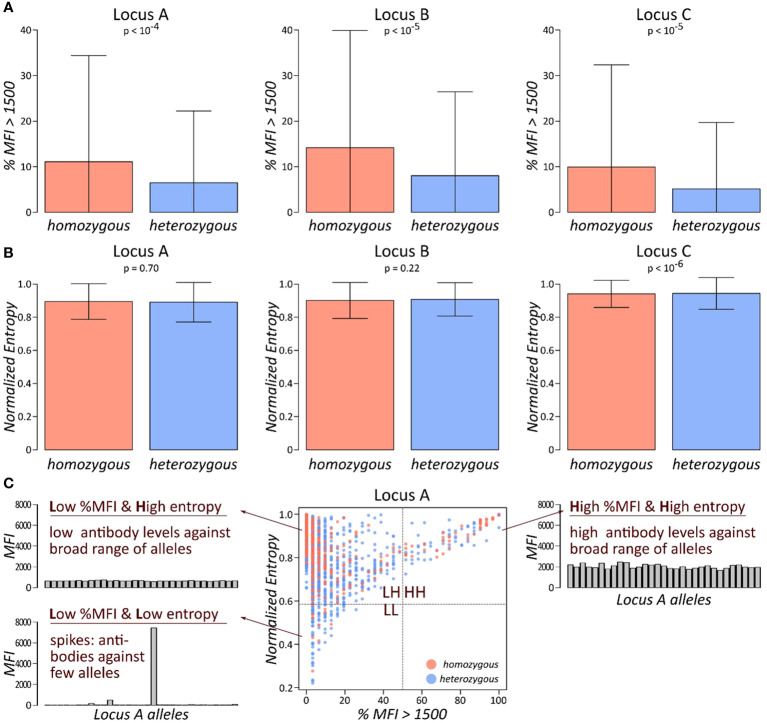
Abundance and specificity of HLA class I antibodies stratified by homozygosity: **(A)** fraction of antibodies with MFI values exceeding 1,500 is shown separately for each class I locus, stratified by homozygous, and heterozygous persons. p-values are derived from Wilcoxon rank-sum test. **(B)** Normalised entropy of antibody profiles of homozygous and heterozygous persons for each locus. p-values are derived from Wilcoxon rank-sum test. **(C)** Each individual’s antibody profile is characterised by a fraction of present antibodies (%MFI>1500) and its normalised entropy, constituting one dot in the biplot of these two characteristics. This biplot is segmented into four quadrants: LL, low %MFI > 1,500 and low entropy; LH, low %MFI > 1,500, and high entropy; and HH, high %MFI > 1,500, and high entropy.

We compared the antibody profiles of homozygous and heterozygous persons as seen by the proportion of antibody beads exceeding MFI of 1,500 (“*%MFI>1,500*”) and by the normalised entropy *H_norm_*. The former is a proxy for the count of HLA antibodies produced by the person; the latter introduces the concept of entropy in the antibody reactivity of a certain locus: a serum was quoted as having a low entropy if the antibodies show mainly low MFI levels with few spike antibodies of very high MFI (see barplots in **Figure 2C**). In contrast, a high entropy profile shows a very uniform MFI distribution. These two antibody parameters were computed separately for each locus and stratified by homozygosity/heterozygosity of the locus. The results for HLA class I loci are presented in **Figure 2**: first, we compared “*%MFI>1,500*” between locus A homozygous and heterozygous persons (**Figure 2A**, left plot). Despite relatively large standard deviations, we found a significant difference between homozygous and heterozygous persons (p-value <10^−4^ in Wilcoxon rank-sum test), indicating that locus A antibodies are more abundant in HLA-A homozygous persons. Similar results were obtained for HLA-B, and C loci (p-values <10^−5^), with significantly increased antibody levels in homozygous persons (**Figure 2A**).

We then compared the second measure for antibody profiles, the normalised entropy. However, no marked difference between homozygous and heterozygous persons could be observed in any of the class I loci (**Figure 2B**). Finally, biplots *%MFI>1,500* versus normalised entropy were generated and segmented into four quadrants (**Figure 2C**). Each quadrant collects individuals with specific antibody profile characteristics: the low *%MFI>1,500* and low entropy quadrant (LL; bottom left) represents persons with mainly low antibody MFI values, complemented by few very abundant antibodies. The low *%MFI>1,500* and high entropy quadrant (LH; top left) collects persons with uniformly low antibody levels. The high *%MFI>1,500* and high entropy quadrant (HH; top right) is characterised by persons with consistently high antibody levels throughout the corresponding alleles. Finally, the fourth quadrant is not populated due to the limitation of antibody levels in the patient’s sera. For each quadrant, one representative antibody profile is shown and briefly characterised (**Figure 2C**).

**Figure 3:**
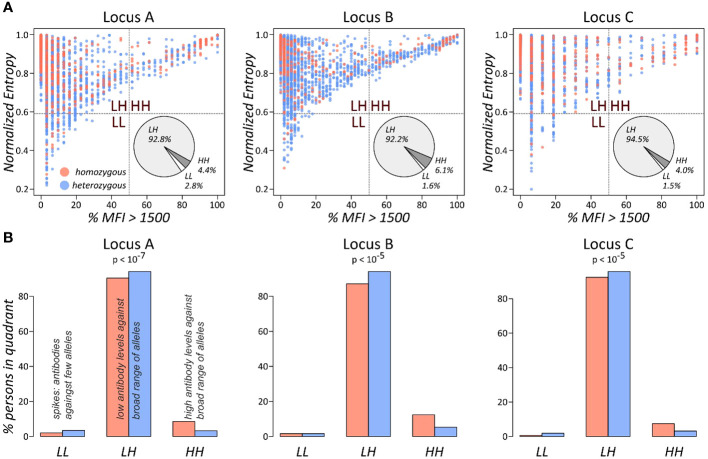
Antibody profile characteristics of class I loci as seen by the % MFI vs. entropy biplots: **(A)** the biplots show the fraction of present antibodies (%MFI > 1,500) and normalised entropy for each person. The pie charts represent overall fractions of persons in each of the quadrants. **(B)** Fractions of persons in the quadrants stratified by homozygous and heterozygous alleles in the locus. p-values were computed using Fisher’s exact test based on the absolute person numbers.

This approach was applied for all three HLA class I loci (**Figure 3**). We found that individuals in the HH quadrant (uniformly high antibody levels against a broad range of HLA alleles) constitute between approximately 4% and 6%, while the vast majority populate the LH quadrant of uniformly lower antibody levels (>90% in the three loci: **Figure 3A**). We then counted numbers of homozygous and heterozygous persons in the populated quadrants, separately for the class I loci. Enrichment statistic revealed highly significant association between homozygosity and number of persons in the quadrants, which is mainly driven by shift from low to high antibody levels in homozygous persons in each of the loci (**Figure 3B**).

These results show that homozygous persons produce significantly more antibodies against a broad range of HLA alleles compared to heterozygous ones.

### 3.3 Class II antibody specificity in homozygous and heterozygous individuals

**Figure 4:**
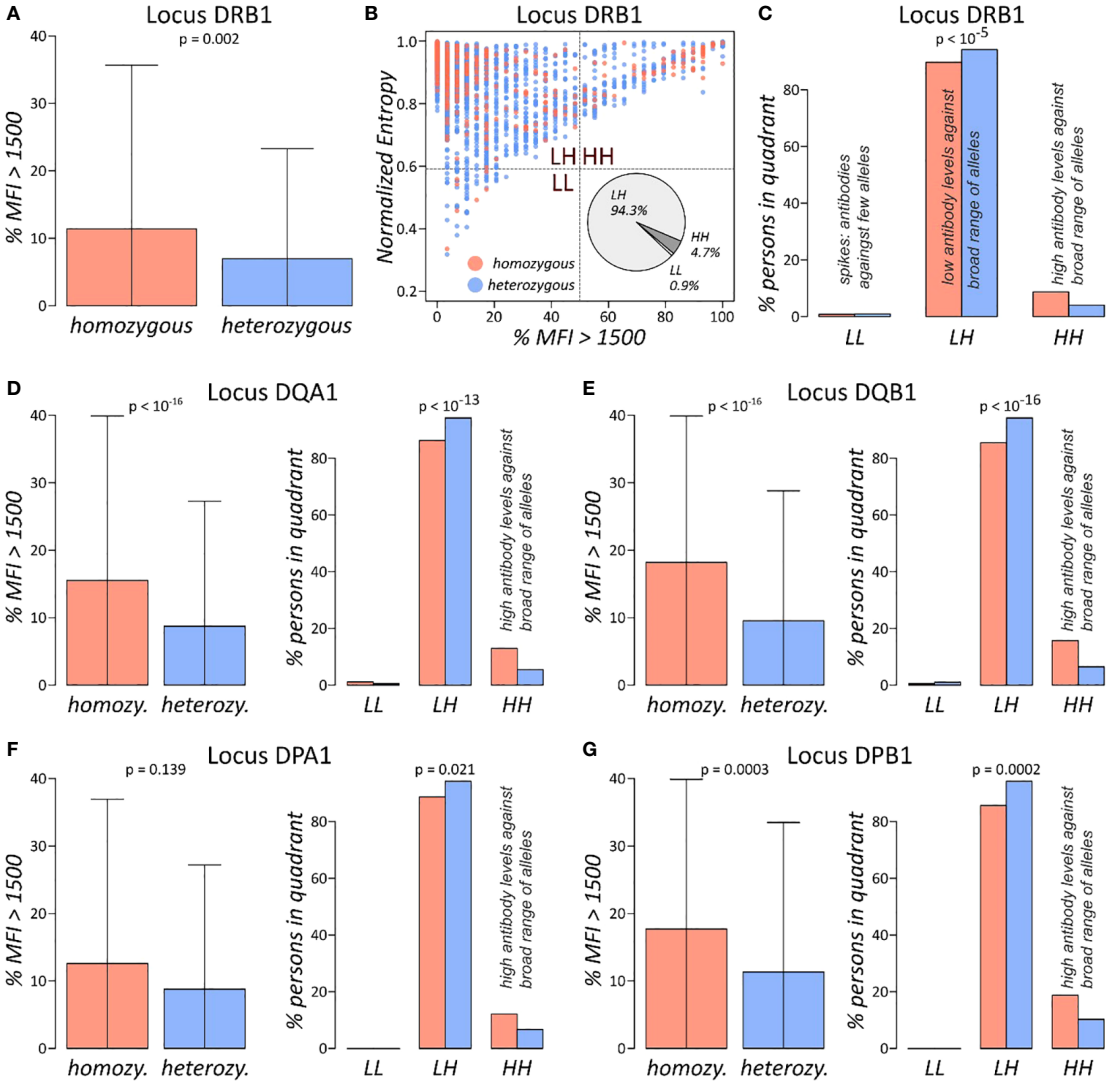
Antibody profile characteristics of class II loci: **(A)** fraction of locus DRB1 antibodies with MFI values exceeding 1,500 separated for homozygous and heterozygous persons. p-value is derived using Wilcoxon rank-sum test. **(B)** The %MFI > 1,500 vs. normalised entropy biplot of DRB1 antibody profiles, together with overall fractions of individuals in each of the quadrants. **(C)** Fractions of individuals in the quadrants stratified by homozygous and heterozygous alleles in the DRB1 locus. p-Value was computed using Fisher’s exact test of the absolute numbers. **(D–G)** %MFI>1,500 plots, and biplot quadrant allocations of the loci HLA-DQA1, DQB1, DPA1, and DPB1, in analogy to **(A, C)**. Corresponding biplots of these loci are shown in **Supplementary Figure 4**.

HLA antibody screening against the class II determinants HLA-DRB1, DQA1, DQB1, DPA1, and DPB1 were analysed in the same manner. For each locus and person, we computed the normalised entropy of the antibody profiles and subsequently compared it between homozygous and heterozygous individuals. As for class I antibodies, entropy is not differential with respect to homozygosity (**Supplementary Figure 3A**). In contrast, the *%MFI>1500* measure shows significant differences for HLA-DRB1, DQA1, DQB1, and DPB1 loci (p-values< 0.002 in Wilcoxon rank-sum test; **Figures 4A, D–G**). DPA1 locus, which is the least polymorphic one with approximately 73% homozygous persons, shows the same trend of higher antibody levels as reflected by *%MFI>1500*, however on a very low significance level (p-value = 0.14).

For all class II loci, biplots of *%MFI>1500* versus normalised entropy were generated (**Figure 4B**; **Supplementary Figure 3B**). Distributions of individuals across the four quadrants is like class I antibody profiling, with majority populating the LH quadrant of low antibody levels against broad range of alleles and approximately 5%–13% in the HH quadrant of uniformly high antibodies. We then tested distribution of the quadrant population numbers stratified by homozygosity status in each locus separately, yielding significant association for all loci (p-values< 0.021 in Fisher’s exact test; **Figures 4C–G**). The analysis showed that homozygous persons increasingly accumulate in the HH quadrant in analogy to class I antigens, meaning that homozygous individuals feature an overall elevated risk for antibody production against a broader spectrum of HLA alleles as compared to heterozygous individuals.

### 3.4 Identification of homozygous risk alleles for antibody production

The previous analyses based on locus-wise homozygosity neglected the particular homozygous allele. We therefore applied our analyses on the allele level, this way evaluating association of homozygosity of an individual allele and the characteristics of the corresponding antibody response/profile. This step increases resolution of our analysis; however, it entails lower numbers of homozygous individuals included in statistical testing. Thus, we discarded alleles with <10 homozygous individuals in the dataset from subsequent antibody specificity profiling.

**Figure 5:**
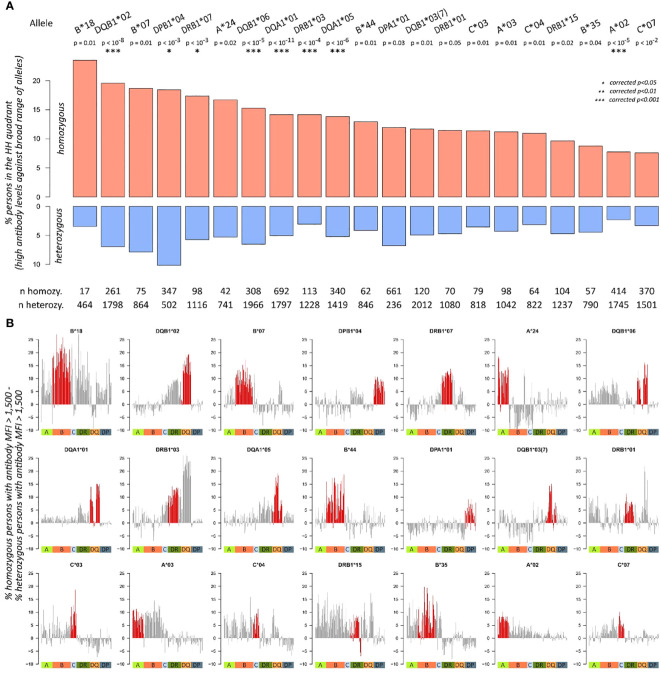
Antibody characteristics of individuals with homozygous and heterozygous alleles: **(A)** proportions of homozygous and heterozygous individuals in HH quadrant (i.e., persons with consistently high MFI values over all antibodies) about individual allele homozygosity. p-Values are derived from Fisher’s exact test; significance levels after Bonferroni correction are highlighted with asterisks. Total numbers of homozygous and heterozygous persons are given below the bar plots. Only alleles with more than 10 homozygous persons in the cohort and p-values< 0.05 are shown here. **(B)** The bar plots show differences in proportion of homozygous and heterozygous individuals producing an antibody (MFI > 1,500) across all antibodies profiled in the study. For each of the 21 significant alleles identified in **(A)**, one bar plot is generated. Red bars indicate antibodies of the same locus as the corresponding homozygosity, the coloured squares below indicate class I, and II antibody loci.

For each of the 43 alleles with more than 10 homozygous individuals in the dataset, we calculated “*%MFI>1500*”, and normalised entropy measures, generated corresponding biplots, and segmented them into the four quadrants as described above. Then, we tested for association of the quadrant populations and homozygosity of the respective allele, obtaining 21 alleles with p-values below 0.05 (**Figure 5**). For these 21 alleles, proportions of individuals in HH quadrant (i.e., persons with consistently high MFI values over all antibodies) are shown for homozygous and heterozygous subgroups (**Figure 5A**). All the alleles revealed an elevated proportion in HH quadrant when comparing homozygous and heterozygous subgroups. These results indicate a skew of specific HLA determinants in a homozygous form in the cohort of patients. This observation would have consequences for homozygous patients. For patients awaiting an organ, an adapted allocation strategy and an appropriate monitoring for the formation of donor-specific antibodies at an early stage should be used.

Until this point, we evaluated only antibody levels of the locus of homozygosity. To check if homozygosity also relates to differential antibody production of other loci, we subtracted the proportions of homozygous and heterozygous individuals producing an antibody (MFI > 1,500) across all antibodies profiled in the study. For an individual antibody, this score is positive; if a higher fraction of homozygous individual produces this antibody, then heterozygous does. This way, we generated antibody profiles of “homozygosity-related elevation” for each of the 21 alleles (**Figure 5B**). It reveals that homozygosity of HLA-B*18 associates to elevated levels of most antibodies in the data, except for parts of the HLA-C and HLA-DQA1 antibodies. Homozygosity of HLA-DQB1*02, in turn, shows increased levels of HLA-DRB3, DQA1, and DQB1 locus antibodies; however, antibodies of the other loci do not show this trend.

**Figure 6:**
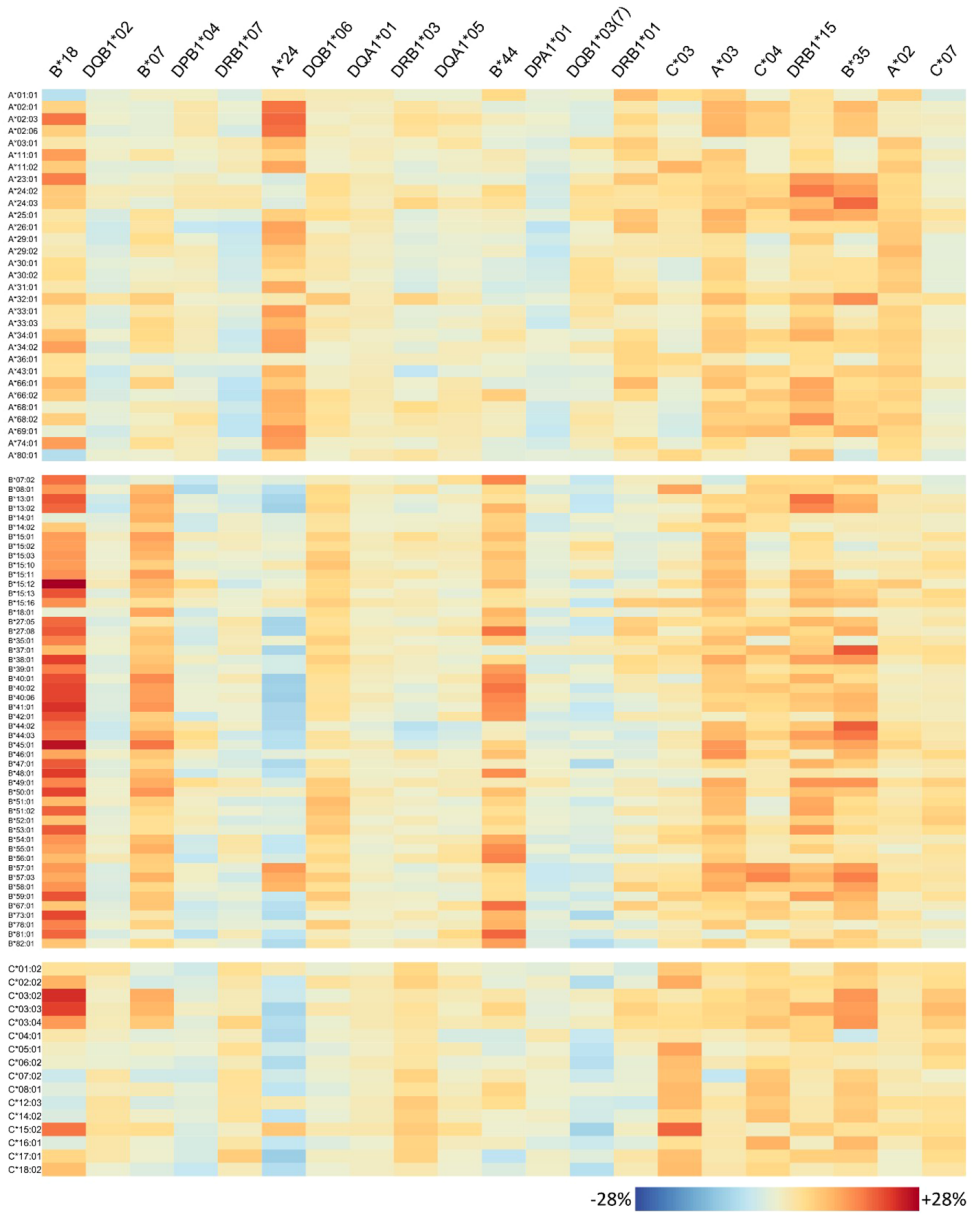
Homozygosity of 21 risk alleles associates with increased abundance of specific HLA-class I antibodies: each column of the heatmap represents a risk allele; rows represent the antibodies. The colours represent percent increase in homozygous persons that produce the antibody compared to heterozygous ones.

The most incident homozygosity in our cohort relates to HLA-DPA1*01 (661 homozygous vs. 236 heterozygous individuals) and shows antibody responses exclusively directed against HLA-DP. Similarly, homozygosity for HLA-A*24 revealed elevated risks for antibody responses against other HLA-A antigens only, whereas homozygosity for HLA-A*03 leads to increased antibody responses against a broader spectrum of HLA-A and HLA-B antigens (**Figures 5**, **6**). This suggests an individual risk depending on the homozygous HLA antigen and not a generalised pattern.

In summary, we identified 21 HLA alleles, which showed a verifiable association of higher antibody levels partly against a broad range of HLA alleles and which can therefore be considered as risk alleles for potential organ recipients.

### 3.5 Allele homozygosity relates to specific antibody patterns

**Figure 7:**
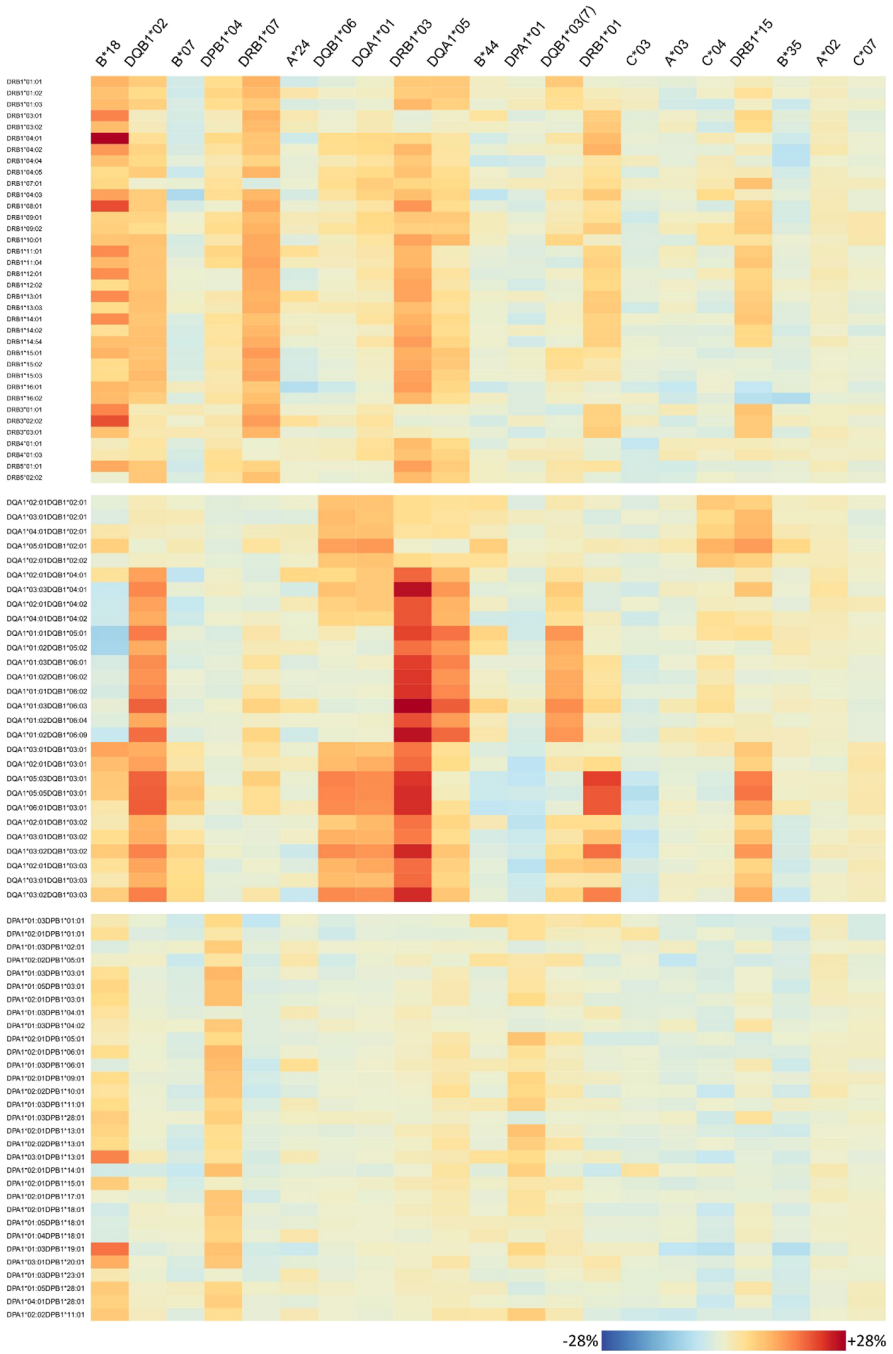
Homozygosity of 21 risk alleles associates with increased abundance of specific HLA-class II antibodies. See legend of **Figure 6**.

The antibody profiles found to be elevated in homozygous individuals mainly show consistently high antibody levels of the corresponding locus, but several outliers can also be observed: some few antibodies of the homozygous locus are not elevated in homozygous persons in the same order of magnitude; however, antibodies belonging to other loci are concertedly increased. To investigate this variability on individual antibody level, we generated heatmaps visualizing the risk alleles as columns and antibodies are rows (**Figure 6** for HLA class I, **Figure 7** for class II).

For each risk allele, the visualisation allows for in-detail inspection of all HLA antibodies either with increased or not increased abundance in homozygous individuals, with potential implications for organ matching. For example, HLA-B*18 homozygosity relates to high antibody levels of HLA-A and HLA-B loci; however, HLA-A*01:01, A*80:01, B*14:01, and HLA-B*18:01 seem not to be elevated (**Figure 6**). HLA-C is more diverse in this respect, showing that especially HLA-C*03:02, C*03:03, C*03:04, and HLA-C*15:02 increased, but also a variety of antibodies do not such as HLA-C*07:02, C*12:03, C*14:02, and HLA-C*16:01. Note that HLA-B*18 homozygosity was also associated with elevation of class II antibodies, in particular majority of DRB1, parts of DQ, and DP alleles tested (**Figure 7**).

Interestingly, homozygosity for HLA class II alleles HLA-DRB1*01 and HLA-DRB1*15 seems to increase the risk for antibody responses against most of the HLA class I antigens (HLA-A, HLA-B, and HLA-C) in contrast to HLA-DQB1*03:01 where a lower risk towards few HLA-A and HLA-B alleles is observed.

## 4 Discussion

In the present study, we concentrated on the analysis of patients being homozygous for any of the tested HLA-A, B, C, DRB1, DQA1, DQB1, DPA1, and DPB1 alleles. Our main objective was the presentation and characterisation of retrospective data collected by the University Hospital Leipzig and the Charité Berlin, in order to understand relations between homozygosity and antibody production relevant for transplantation immunology. Therefore, we evaluated the antibody repertoires of the individuals in our data set using a stepwise analysis workflow. Our results support the immunological hypothesis that homozygosity leads to an increase in presentation of specific peptides, which might lead to an increase in antibody production. In turn, this has strong implications for immunogenicity in the context of transplantation and organ rejection. A subsequent study on this question requires further clinical information that we are currently gathering and will be presented in a later publication.

As stated before, we did not include the products of HLA-DRB3, DRB4, and DRB5 because they are in a tight linkage disequilibrium with the DRB1 counterparts. Furthermore, we could not include individual immunising events into our analyses because information on these events was only partly surveyed during sample acquisition, potentially missing events not in the context of transplantations such as previous diseases, pregnancies, and abortions.

Another limitation is the use of the semi-quantitative MFI values, which are established to categorise an antibody as “present” or “absent” based on the cutoff value recommended by the manufacturer (MFI > 1,500), however without a calibrated absolute scale. This drawback is circumvented in our antibody profiling by the two measures used: the “%MFI > 1,500” measure is based on the threshold-based dichotomisation into “present” and “absent” antibodies, which is the primary scope of the test kits. Normalised Shannon entropy, in turn, is invariant with regard to proportional shift of the MFI values possibly due to systematic bias of the measurement process. Furthermore, this entropy involves logarithmised values, potentially compensating moderate, bead-specific shifts. Finally, entropy values of a locus are used in our study in direct pairwise comparisons (homozygous vs. heterozygous individuals), mitigating outlying beads and generally avoiding the effect of locus-specific shifts.

In the “think different” cohort, we analysed the occurrence of homozygosity for single genes for all classical HLA gene loci (MHC complex). As expected, the degree of homozygosity differed from locus to locus, which can be explained by the frequency of the determinants within the locus following the rule that the degree of polymorphism within the locus dictates the degree of homozygosity. This means that the higher the degree of polymorphism, and the more evenly distributed among a locus, the lesser the degree of homozygosity, which holds exemplarily true for HLA-B. The HLA locus DPA1 is the counter example with a very limited count of alleles and a significantly skewed distribution towards the most abundant allele HLA-DPA1*01, which results in a substantial degree of homozygosity for HLA-DPA1*01 in our cohort. Homozygosity, termed as the availability of two identical alleles or allele groups in a patient, is seen significantly associated with the ability to produce alloantibodies triggered by an alloimmunising stimulus (transfusions, pregnancy, and/or transplantation). This can be explained by the fact that homozygous individuals in general show a markedly reduced count of HLA determinants (i.e., epitopes) against, which an individual cannot induce an alloimmune reaction. Conversely, these homozygous individuals can be triggered by a broader range of HLA antigens and determinants/epitopes, resulting in an increased risk for antibody responses against a broader spectrum of HLA antigens even across loci. It is well accepted that there is no association of specific HLA determinants with the fact of being a patient awaiting an organ reported so far, besides some renal diseases such as HUS, IgA nephropathy (28, 29). Homozygosity for HLA-A, HLA-B, and HLA-DRB1 are usually prioritised in different allocation schemes worldwide; however, the patient being homozygous in one of the clinically relevant eight other loci is rather neglected, although they tend to produce more specific antibodies (4, 12). Furthermore, the data presented above show a direct correlation of homozygous loci (HLA-A, HLA-B, HLA-C, HLA-DRB1, HLA-DQA1, HLA-DQB1, HLA-DPA1, and DPB1) to antibody production and, moreover, of variants to antibody production towards other alleles of the same locus. For pregnancies, Hönger et al. elaborated on the frequency determination of pregnancy-induced alloantibodies (11). A subgroup of homozygous mothers tended to frequently produce more antibodies. Admittedly, the presented numbers were quite low. These data resembles the results reported earlier in several contributions in that specific mismatch led to graft losses (20, 30). In our data presented above, we concentrated not only on the degree of homozygosity, depicted as the number of loci being homozygous, but also on the patient-specific homozygous alleles in the loci typed. To our opinion, not only the number of homozygous genes is important, but also the specific patient–donor mismatch plays a crucial role in the sensitisation and later on graft survival.

Therefore, we termed the concept leading to the information reported here as the taboo concept 2.0. For the sake of the patients, we propose that in case of homozygosity in either of the HLA loci, organ allocation should be patient specific with immunologically compatible organs with respect to the risk of a *de novo* immune response. We are indeed aware that this cannot be achieved in every case. Making use of possible neutral mismatches might help. The present study was performed on a cohort of patients from two transplantation centres and local or organ donors from one allocation organisation. It is therefore imperative to repeat the study with different populations and different predominant haplotypes.

In conclusion, the concept of homozygosity assessment, “taboo 2.0”, allowed us to determine the influence of a specific mismatched allele on antibody formation, while the use of heterozygous situations might jeopardise the results.

## Data availability statement

Individual data on antibody abundances and homozygosity is provided as **Supplementary Table 1**. Full genetic data cannot be directly provided because of ethical and privacy restrictions (Genetic Diagnostics Act, Germany). Requests to access this data should be directed to c.lehmann@medizin.uni-leipzig.de.

## Ethics statement

The studies involving humans were approved by Ethik-Kommission, Medical Faculty University Leipzig (336/23). The studies were conducted in accordance with the local legislation and institutional requirements. The participants provided their written informed consent to participate in this study.

## Author contributions

HL-W: Formal analysis, Investigation, Methodology, Software, Validation, Visualization, Writing – original draft, Writing – review & editing. CL: Conceptualization, Data curation, Investigation, Methodology, Project administration, Resources, Supervision, Validation, Writing – original draft, Writing – review & editing. NL: Data curation, Investigation, Methodology, Project administration, Resources, Supervision, Writing – review & editing. ID: Conceptualization, Investigation, Project administration, Resources, Supervision, Validation, Writing – review & editing.

## Supplementary material

Supplementary Figure 1 Schematic of the analysis workflow. **(A)** Class I antibody data is available for 4,515 individuals, class II antibodies for 5,793 individuals (overlap of 3,074 individuals). An individual’s MFI values across the beads represent the corresponding ‘antibody profile’, which is further divided into locus-wise antibody profiles in subsequent analyses. **(B)** Immunogenetic typing is available for 65,169 individuals. 29,581 of them were typed in high-resolution, the remaining 35,588 individuals in low-resolution. A locus is regarded as homozygous, if both alleles code for the same antigen group. **(C)** Locus-wise antibody profiles are evaluated in terms of overall abundance and specificity using the two measures ‘%MFI>1,500’ and ‘normalized entropy’, respectively. **(D)** For each individual, both measures are computed and plotted into a two-dimensional coordinate system, using ‘%MFI>1,500’ as x-coordinate, and ‘normalized entropy’ as y-coordinate. Each dot in this biplot represents one individual person. **(E)** The biplot is segmented into four quadrants, with the three populated quadrants (termed LL, LH, and HH) showing characteristic antibody profiles as indicated by the example profiles depicted. **(F)** Distribution of all individuals in the biplot across the quadrants is visualized in terms of a pie chart (left part), or as barplots grouped according to homozygosity of the individuals (right part). Individuals in the HH quadrant show high antibody levels against a broad range of alleles, thus being at risk for organ rejection. Therefore, prevalence of the HH quadrant is screened in individuals with different homozygous alleles and shown side by side in a barplot (part below).

Supplementary Figure 2 Overview of available typing data per locus and homozygosity prevalence. **(A)** Number and fraction of persons with available HLA typing per locus. **(B)** Fractions of homozygous and heterozygous persons regarding each locus. The left bar involves all persons in the cohort, the right bar restricts to persons with additional antibody (LS) profiling performed, respectively. Absolute number of persons with LS data is given below the bars.

Supplementary Figure 3 Additional antibody profile characteristics of class II loci. **(A)** Normalized entropy of antibody profiles of homozygous and heterozygous persons for each locus. p-values are derived from Wilcoxon rank-sum test. **(B)** Biplots of the fraction of present antibodies (%MFI>1500) and normalized entropy for each person. The pie charts represent overall fractions of persons in each of the quadrants.

Supplementary Figure 4 Antibody profile characteristics of persons with homozygous and heterozygous alleles. Only alleles with more than 10 homozygous persons in the cohort are assessed. **(A)** Biplots of the fraction of present antibodies (%MFI>1500) and normalized entropy for each person, stratified by homozygous and heterozygous persons. **(B)** Fraction of persons in HH quadrant for each allele. p-value levels are indicated by asterisks.

Supplementary Table 1 Individual data on antibody abundances and matched locus-wise homozygosity information.
