# Supplementary figures and images for: Homozygosity in any HLA locus is a risk factor for specific antibody production: the taboo concept 2.0
Source: Front Immunol. 2024 May 22;15:1384823. doi: 10.3389/fimmu.2024.1384823 (PMC11150536; doi:10.3389/fimmu.2024.1384823)

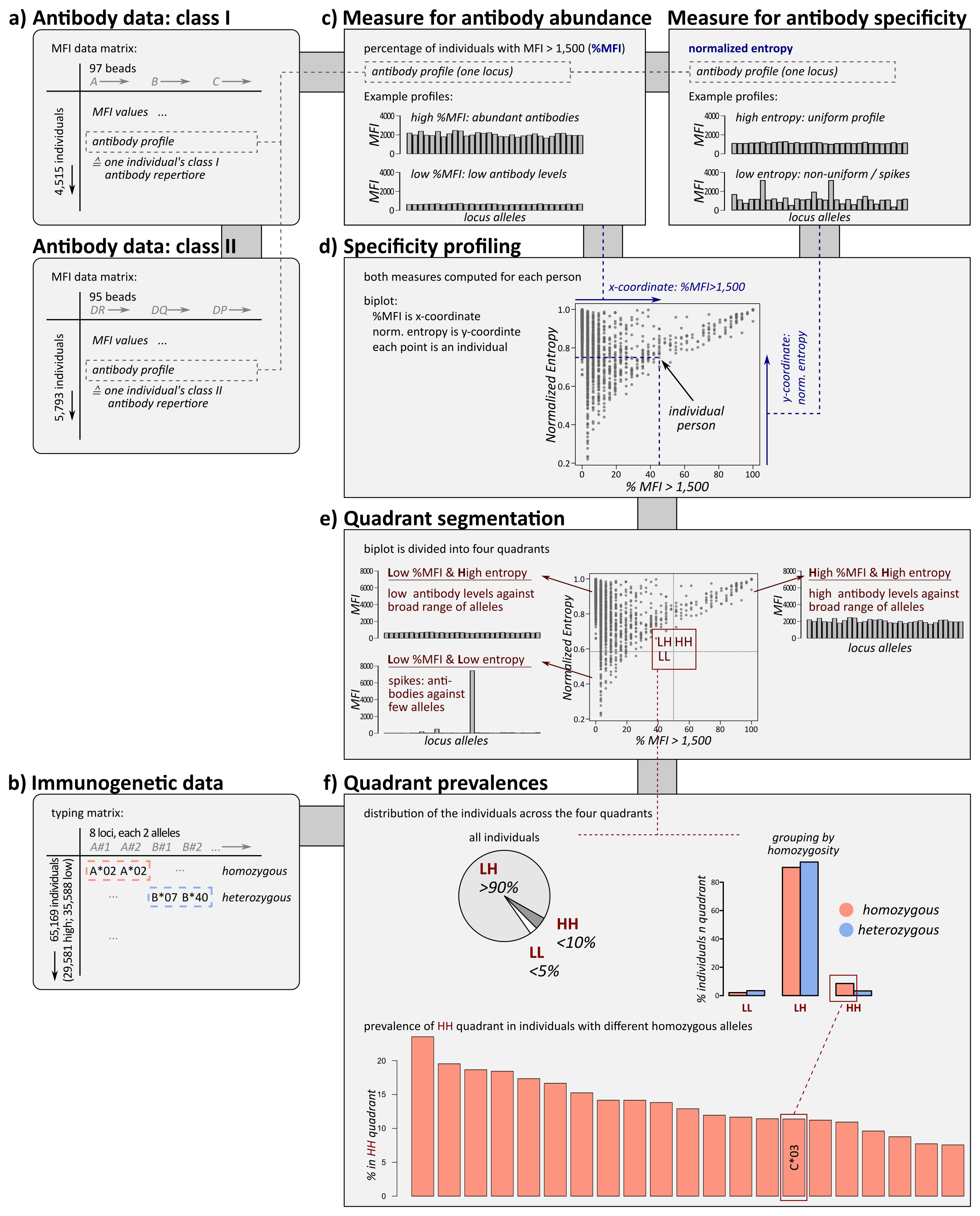

Supplement: Supplementary Figure 1 — Schematic of the analysis workflow. (A) Class I antibody data is available for 4,515 individuals, class II antibodies for 5,793 individuals (overlap of 3,074 individuals). An individual’s MFI values across the beads represent the corresponding ‘antibody profile’, which is further divided into locus-wise antibody profiles in subsequent analyses. (B) Immunogenetic typing is available for 65,169 individuals. 29,581 of them were typed in high-resolution, the remaining 35,588 individuals in low-resolution. A locus is regarded as homozygous, if both alleles code for the same antigen group. (C) Locus-wise antibody profiles are evaluated in terms of overall abundance and specificity using the two measures ‘%MFI>1,500’ and ‘normalized entropy’, respectively. (D) For each individual, both measures are computed and plotted into a two-dimensional coordinate system, using ‘%MFI>1,500’ as x-coordinate, and ‘normalized entropy’ as y-coordinate. Each dot in this biplot represents one individual person. (E) The biplot is segmented into four quadrants, with the three populated quadrants (termed LL, LH, and HH) showing characteristic antibody profiles as indicated by the example profiles depicted. (F) Distribution of all individuals in the biplot across the quadrants is visualized in terms of a pie chart (left part), or as barplots grouped according to homozygosity of the individuals (right part). Individuals in the HH quadrant show high antibody levels against a broad range of alleles, thus being at risk for organ rejection. Therefore, prevalence of the HH quadrant is screened in individuals with different homozygous alleles and shown side by side in a barplot (part below). [file Image_1.png]

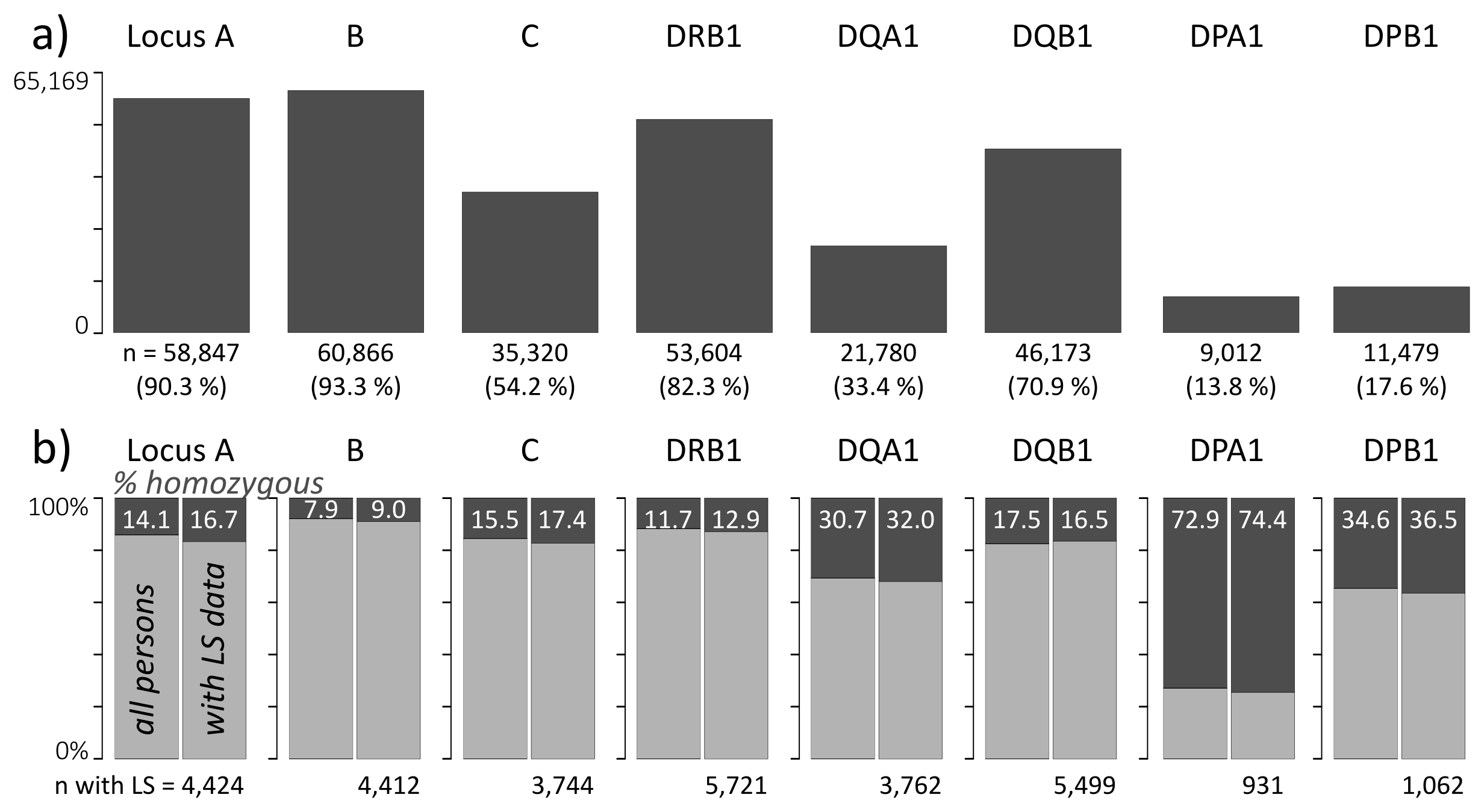

Supplement: Supplementary Figure 2 — Overview of available typing data per locus and homozygosity prevalence. (A) Number and fraction of persons with available HLA typing per locus. (B) Fractions of homozygous and heterozygous persons regarding each locus. The left bar involves all persons in the cohort, the right bar restricts to persons with additional antibody (LS) profiling performed, respectively. Absolute number of persons with LS data is given below the bars. [file Image_2.png]

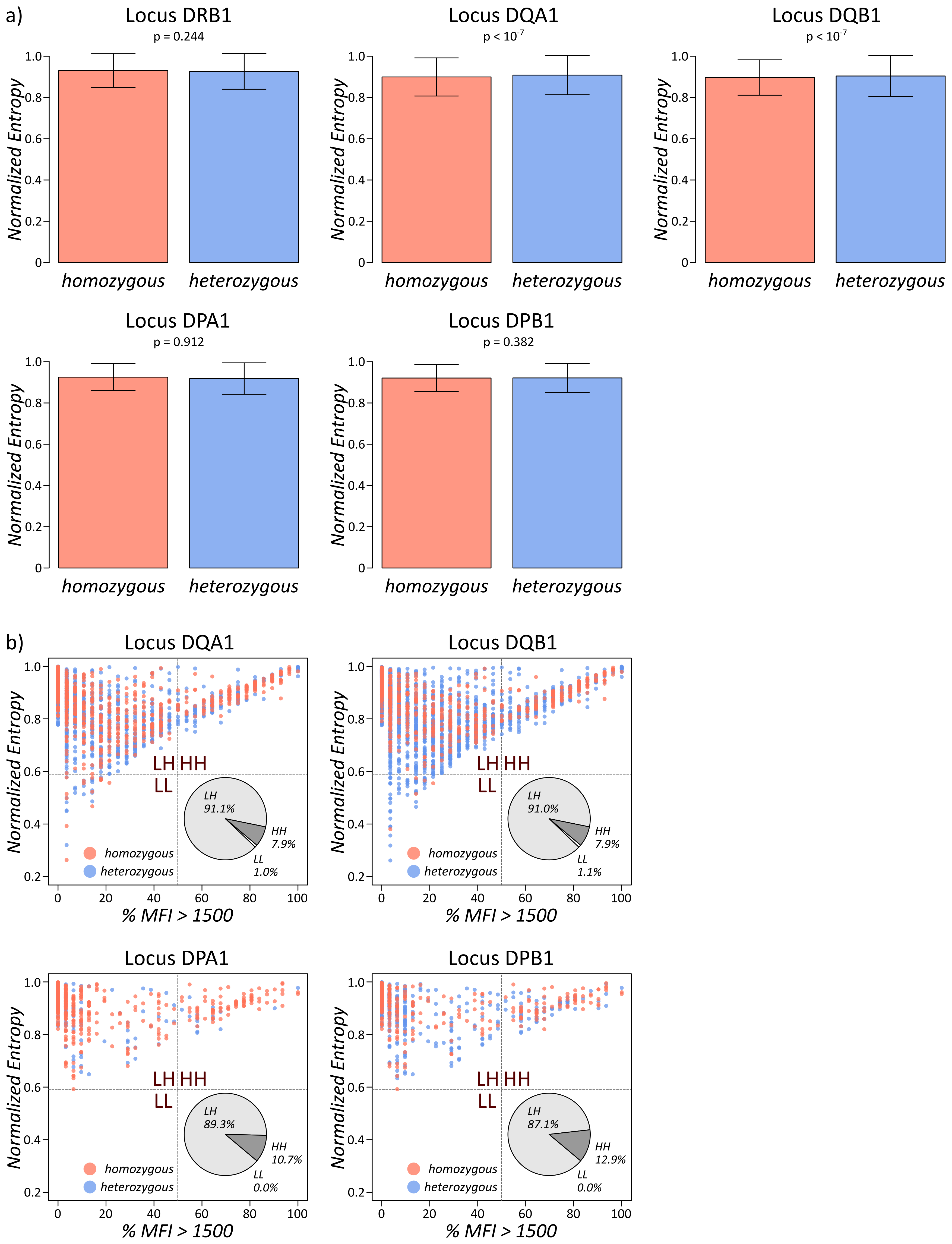

Supplement: Supplementary Figure 3 — Additional antibody profile characteristics of class II loci. (A) Normalized entropy of antibody profiles of homozygous and heterozygous persons for each locus. p-values are derived from Wilcoxon rank-sum test. (B) Biplots of the fraction of present antibodies (%MFI>1500) and normalized entropy for each person. The pie charts represent overall fractions of persons in each of the quadrants. [file Image_3.png]

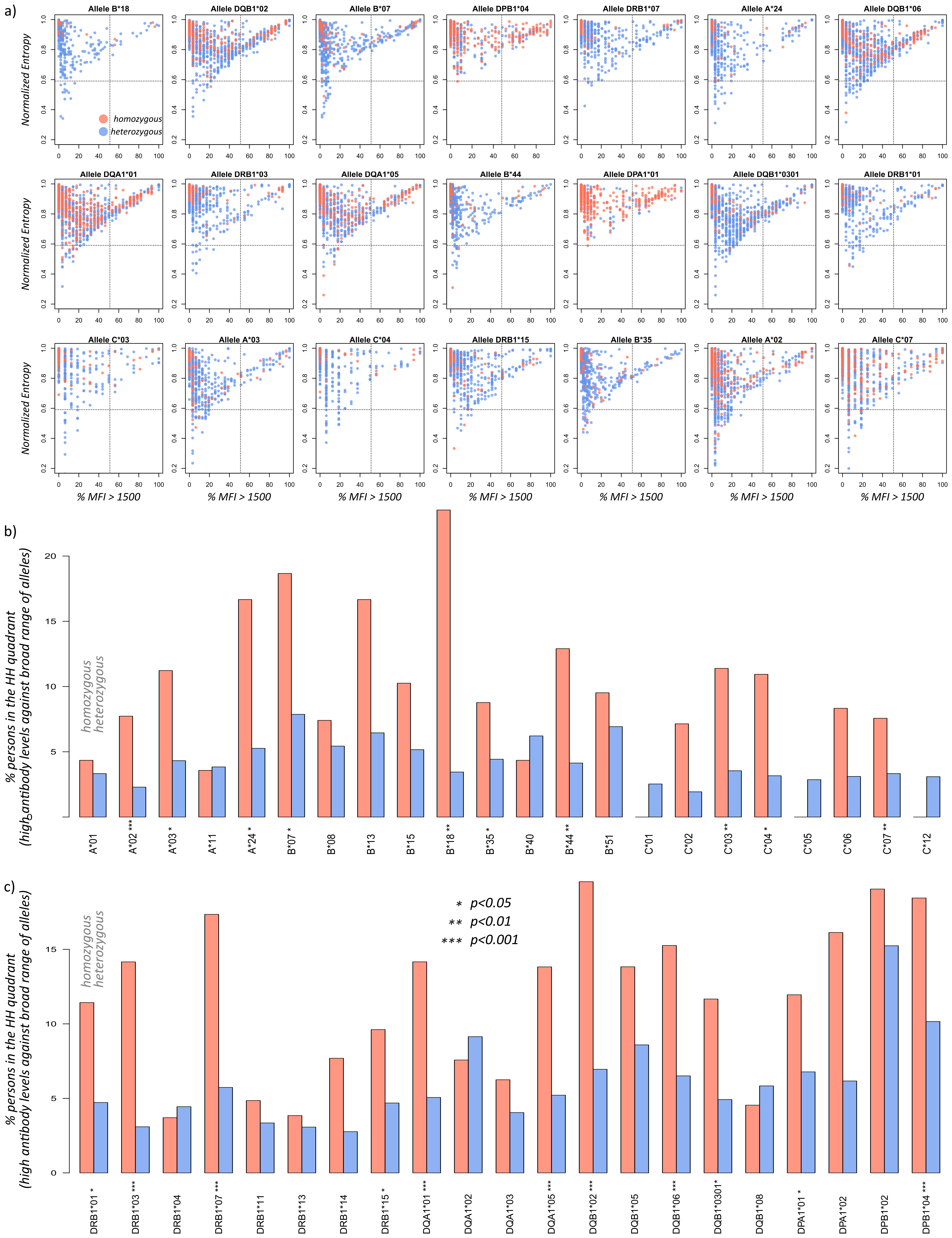

Supplement: Supplementary Figure 4 — Antibody profile characteristics of persons with homozygous and heterozygous alleles. Only alleles with more than 10 homozygous persons in the cohort are assessed. (A) Biplots of the fraction of present antibodies (%MFI>1500) and normalized entropy for each person, stratified by homozygous and heterozygous persons. (B) Fraction of persons in HH quadrant for each allele. p-value levels are indicated by asterisks. [file Image_4.png]
